# Vancomycin-Soaked Quadriceps Tendon Autografts but Not Hamstring Tendon Autografts Are Associated with Early Graft Failure (Graft Resorption) After Primary Anterior Cruciate Ligament Reconstruction: A Single-Center Experience

**DOI:** 10.1007/s43465-025-01632-x

**Published:** 2026-01-03

**Authors:** Patrick Weninger, Veronika Weninger, Andreas Herbst

**Affiliations:** Sports Medical Center, Am Hof 11/9, 1010 Vienna, Austria

**Keywords:** Anterior cruciate ligament, Vancomycin, Graft resorption, Hamstring tendon autograft, Quadriceps tendon autograft

## Abstract

**Introduction:**

Anterior cruciate ligament (ACL) injuries are among the most common orthopedic injuries. While Hamstring Tendon (HT) autograft reconstructions have been the most frequent graft type over the last decade, quadriceps tendon (QT) autografts have become increasingly popular in the past 2 years. The procedure of vancomycin graft soaking has become the state-of-the-art in many institutions as it avoids septic knee arthritis after anterior cruciate ligament reconstruction (ACLR). In the present study, we analyzed all patients presenting with an ACL rupture who subsequently underwent primary ACLR using either HT or QT autografts soaked in vancomycin during a 12-month study period. We specifically focused on the occurrence of graft resorption leading to early graft failure, comparing outcomes between HT and QT autografts.

**Materials and Methods:**

Relevant data of all patients presenting with a primary anterior cruciate ligament (ACL) rupture and subsequently undergoing ACL reconstruction (ACLR) using either a hamstring tendon (HT) or quadriceps tendon (QT) autograft were collected between April 2022 and April 2023. Patients who later presented to our outpatient clinic with clinical signs of recurrent knee instability, with or without a preceding trauma, underwent magnetic resonance imaging (MRI) to evaluate graft integrity.

Early graft failure was defined as recurrent instability within 12 months after the index ACLR, confirmed by MRI evidence of an absent or nonfunctional graft, and—if revision surgery was performed—arthroscopic confirmation of graft absence.

**Results:**

A total of 468 patients underwent anterior cruciate ligament reconstruction (ACLR) using autografts, including 421 hamstring tendon (HT) and 47 quadriceps tendon (QT) grafts. During the 12-month follow-up period, early graft failure occurred in 22 patients (all QT autografts), corresponding to a failure rate of 46.8% in the QT group and 0% in the HT group. Nineteen of the affected patients underwent revision ACLR, while three declined further surgery.

**Discussion:**

The markedly higher rate of early graft failure in the QT group raises concern regarding the safety of combining vancomycin soaking with quadriceps tendon autografts. These findings should be interpreted with caution and considered hypothesis-generating. Further prospective and experimental studies are required to confirm these observations and clarify the underlying mechanisms.

## Introduction

Anterior cruciate ligament (ACL) injuries represent one of the most common orthopedic conditions, with an estimated 250,000 ACL reconstructions performed annually in the United States [1]. It is common knowledge that an ACL rupture leads to knee instability and to impaired knee biomechanics [2]. In active patients and athletes, ACL reconstruction (ACLR) is the preferred treatment option to restore knee stability and to avoid secondary damage to menisci and cartilage which may otherwise lead to long-term joint degeneration or osteoarthritis [3]. In selected patients, conservative treatment might be feasible [4, 5]. Surgical options include ACLR using autologous tendon grafts (hamstring tendons HT, quadriceps tendon QT, patellar tendon PT), allografts (donor tendons), or methods for preserving the ACL. All mentioned graft types have pros and cons in terms of donor-site morbidity, mechanical strength, re-rupture rates, or infection rates [6]. Whereas HT autografts have been the most frequently used graft type for ACL reconstructions during the last decade, QT autografts have become increasingly popular within the past 2 years. This may be related to easier harvesting and also to reports indicating superiority to HT autografts in terms of re-rupture rates [7]. Harvesting the QT autograft is easy, does not compromise hamstring strength, and also spares the medial side in case of medial collateral ligament injuries [8]. As a result of the aforementioned advantages, QT autografts became the graft of choice not only for revision ACLR, but also for primary ACLR for more and more surgeons [7]. Recently, the antibiotic vancomycin has gained wide attention in ACLR as large studies pointed out beneficial effects of vancomycin on infection rates after ACLR. Vancomycin soaking is typically performed by immersing the graft in a vancomycin solution prior to implantation, which is thought to act locally to prevent bacterial colonization. The procedure of vancomycin graft soaking has become state-of-the-art in many institutions as it avoids septic knee arthritis after ACLR [9]. Concerns have been raised that vancomycin might compromise the biological tendon environment and/or also cause toxic damage to the knee cartilage. So far, these concerns could not be verified, and vancomycin seems to be a safe and effective method reducing infection after ACLR [10]. In this present investigation, we assessed all patients presenting with an ACL rupture and subsequently undergoing primary ACLR either with HT or QT autografts soaked in vancomycin during a 12-month period. We hereby focused on the phenomenon of graft resorption leading to early graft failure in comparison between HT autografts and QT autografts. This retrospective study aimed to compare the incidence of early graft failure due to resorption in patients undergoing primary ACLR using vancomycin-soaked QT versus HT autografts over a 12-month period.

## Materials and Methods

The study was conducted in accordance with the ethical principles of the Helsinki Declaration and approved by the Ethics Committee of the City of Vienna (protocol number EK23-052-VK). All participants provided informed consent for data collection and use in this study.

Relevant data were collected from all patients presenting with primary ACL rupture who underwent ACLR with either HT or QT autografts between April 2022 and April 2023. All primary ACLR grafts were soaked in 1% vancomycin for 15 min after graft preparation. All patients were either operated by the first author or were referred from other institutions for further treatment after undergoing ACLR. Patients referred from other institutions were included only if operative reports confirmed that their index procedure was a primary ACL reconstruction and that the identical vancomycin-soaking protocol (1 % solution for 15 minutes) was used. Cases lacking such documentation or representing revision ACLR were excluded.

Demographic information and any concomitant injuries were recorded for all patients. The protocol for ACLR and for the postoperative rehabilitation was recently described in a study by Weninger et al. Patients were followed up every 2 months for at least 12 months at our clinic. Episodes of recurrent instability—whether traumatic or atraumatic—or clinical signs of knee instability (Lachman test) prompted MRI evaluation and/or revision ACLR. Graft integrity was evaluated by the same senior radiologist (V. P.-W.) in all cases and confirmed arthroscopically during revision surgery. Early graft failure was defined as recurrent knee instability confirmed by absent graft on MRI and arthroscopy, resulting in revision ACLR within 12 months of the primary surgery. A Pearson Chi-square test was used to compare the frequency of early graft failure between QT and HT autograft groups. A *p* value < 0.05 was considered statistically significant. The analysis was performed using standard statistical software.

## Results

During the study period, our institution was consulted by 468 patients after sustaining an ACL rupture amenable to primary ACLR and receiving surgical treatment. Of these, 424 patients were operated on by the first author, while 44 patients were operated at other institutions and consulted our institution for further treatment.

In 421 patients, HT autograft ACLR was performed, and 47 patients underwent QT autograft ACLR. During the 12-month follow-up period, early graft failure was recorded in 22 patients, all belonging to the QT group. Revision surgery was performed in 19 patients, whereas 3 patients refused further revision surgery. MRI scans were available in all patients.

Early graft failure was documented in 0/421 patients after HT autograft ACLR and in 22/47 (46.8%) patients after QT autograft ACLR during the study period. This difference was highly statistically significant (*χ*^2^ = 196.47, *p* < 0.0001), indicating a strong association between QT autograft use and early graft failure. No case of septic arthritis was observed in either group, confirming the expected protective effect of vancomycin against infection and making postoperative infection an unlikely cause of the observed graft resorption.

**Fig. 1:**
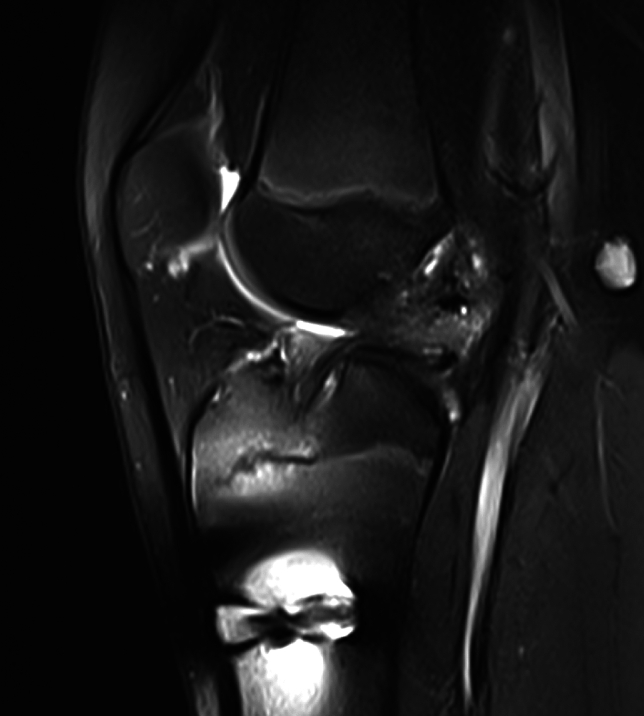
16-year-old male patient at follow-up MRI 5 months after QT autograft ACLR showing complete graft resorptionand missing graft signal 5

**Fig. 2:**
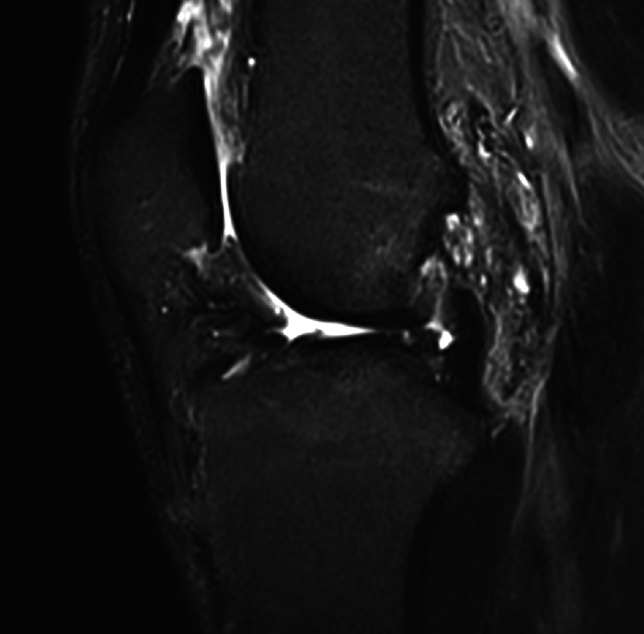
31-year-old male patient at follow-up MRI 7 months after QT autograft ACLR showing complete graft resorption and missing graft signal 6

**Fig. 3:**
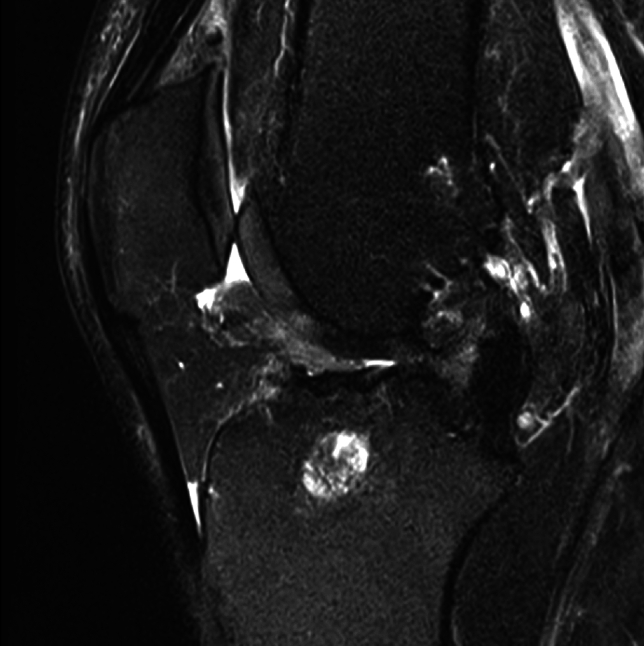
42-year-old female patient at follow-up MRI 7 months after QT autograft ACLR showing complete graft resorption and missing graft signal 7

Figures 1, 2, 3 show MRI scans of representative patients with early graft failure after QT autograft ACLR

**Fig. 4:**
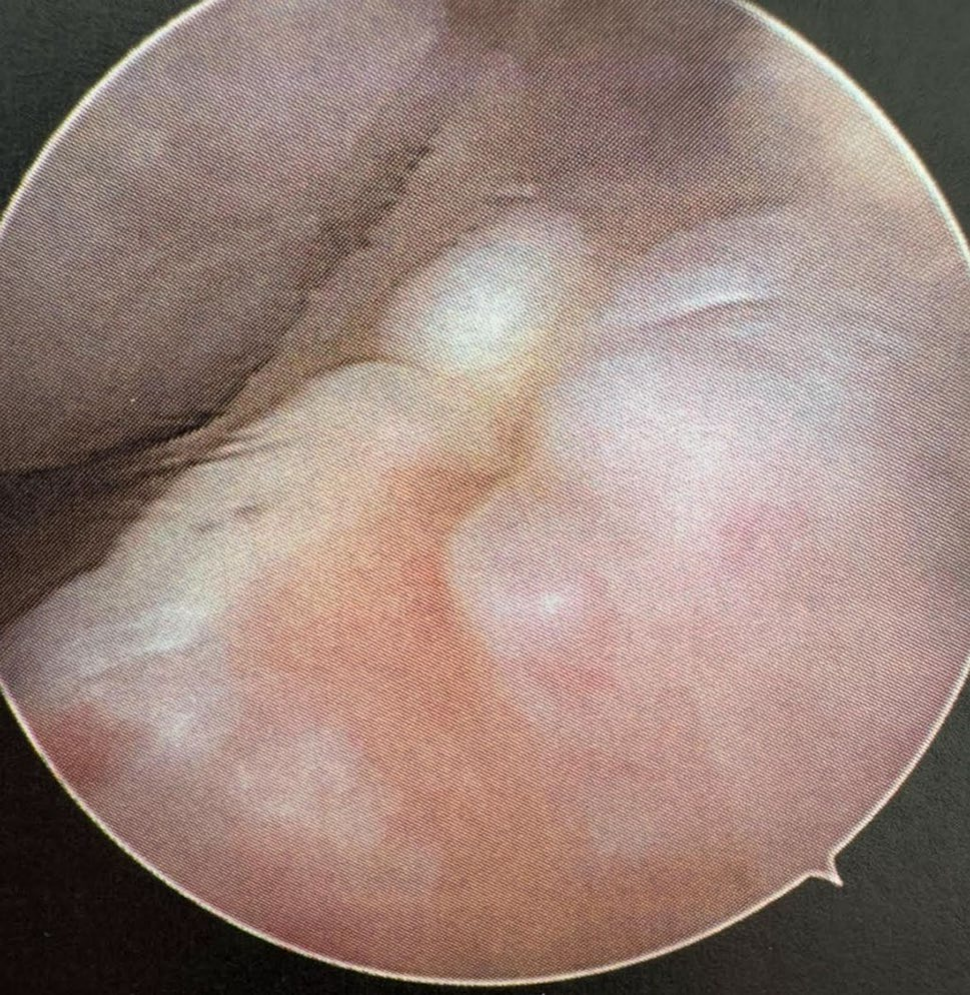
Arthroscopic view into the empty notch of the patient of Fig. 1 showing the missing 95 QT autograft with only a little tibial remnant visible. The graft is completely resorbed 8

Figure 4 shows the arthroscopic appearance of complete graft resorption.

In all revision cases within the QT group, the graft was found to be completely resorbed on arthroscopic inspection, showing no evidence of a true re-rupture or residual tendon remnants. None of the patients in the study population had a history of septic arthritis.

## Discussion

ACLR with tendon autografts is an established surgical method to restore knee anatomy and mechanical integrity after ACL injury [1]. To date, typical graft choices are HT, patellar tendon, and QT autografts. In addition, allografts can be used to replace the torn ACL [6]. Each graft type has specific advantages and disadvantages, and the final choice typically depends on the surgeon’s experience and personal preference [11]. The superiority of one graft over the other has been the main topic of numerous publications and trials [5–7, 12]. So far, the optimal graft choice would be one that has low donor-site morbidity, is easy to harvest, possesses solid mechanical properties, and provides reliable graft integrity over a long period with a comparably low re-rupture rate. Overall, the ideal graft type should reliably avoid secondary damage to intraarticular structures and also should preserve the cartilage and lower the risk of post-traumatic osteoarthritis [13]. The main rationale of using the middle third of the QT for ACLR is clear: It is easy to harvest under direct vision which makes it also accessible to inexperienced surgeons with low caseload [6, 7]. The use of QT autografts for ACLR has been first described by Chen et al [14]. The tendon can either be used as a bone-free soft tissue or connected to a patellar bone block [8]. Several recent studies have reported comparable patient-reported outcome scores, similar re-rupture rates, and lower donor-site morbidity following ACLR with QT autografts compared to HT autografts. Mouarbes D et al. showed comparable outcomes after using QT autografts for ACLR when compared to patellar tendon or HT autografts but also found significantly less harvest site pain after QT use [15]. This is in accordance with Dai W et al and also with Sim K et al [13, 16]. Cavaignac E et al. demonstrated that the use of a QT autograft leads to equal or better functional outcomes than does the use of an HT graft, without affecting morbidity [12]. Runer et al. reported similar clinical results comparing QT and HT tendon autografts in a 2 year patient-reported outcome study [17]. In a very recent study, Raj S. et al. concluded that there was no difference between QT and HT in patient-reported outcome, graft failure rates, or overall complications [18]. However, most studies comparing outcome data of QT and HT autografts focus on patient-reported parameters and do not specify whether vancomycin was used or how its use might have influenced the results [7, 15]. Phegan et al. were the first who report on 1300 patients after ACLR and using vancomycin to reduce infection rate to zero [19]. This is why Eriksson et al. described this procedure as modern rationale for morbidity prevention and patient safety [20]. Pre-soaking the autograft using vancomycin today has become a common practice to drastically reduce postoperative septic arthritis. To date, numerous studies provide reliable data on this topic and highlight the efficacy and safety of this routine in ACLR surgery [10, 21]. Some authors pointed out possible negative effects of vancomycin on the tendon graft as effect of its cytotoxicity and mentioned the unclear mechanism of graft failure of the graft after vancomycin exposure. To date, all of the studies focusing on the potential cytotoxicity of vancomycin revealed no adverse outcome after using it for graft soaking [10, 22]. A major limitation of the current literature, however, is that in nearly all studies reporting on vancomycin in ACLR, HT autografts were the predominant graft type. In fact, there are only a few studies including QT autografts at all. Offerhaus C. published a study with more than 95% HT autografts and 3% QT autografts [21]. In a study by Xiao M., 94.5% of the grafts were HT autografts [23]. Jahier et al. showed comparable stability of knees reconstructed with vancomycin-soaked ACL autografts to contralateral knees with intact ACL. Again, the rate of HT autografts in this study was 100% [22]. This is in accordance with Banios K et al. who also found that septic arthritis could be eliminated using vancomycin in HT autografts but did not include patients undergoing ACLR with QT autografts [24]. To date, no clinical or experimental study has specifically evaluated the effects of vancomycin on the fate of QT autografts. Atherton et al. demonstrated that vancomycin not only does not alter the molecular structure of the ACL hamstring graft but moreover does improve its integrity [25]. Xiao M et al. showed that tenocytes derived from human patellar tendons exposed to relatively high concentrations of vancomycin for short periods of time do not demonstrate significant cell death and toxicity. However, comparable experimental or molecular investigations for QT autografts do not yet exist [23]. Graft soaking in vancomycin has been shown to be an effective and safe procedure when using HT autografts. It eliminates septic arthritis after ACLR and improves patient safety to a maximum [21]. According to both the existing literature and the results of the present study, these results cannot yet be extended to QT autografts for ACLR. In light of our findings and current literature, we emphasize the need for clinical trials and experimental studies to investigate the effects of vancomycin on QT autografts. We hypothesize that the cellular and microstructural properties of a QT autograft are different from those of HT autografts and might make the QT autograft more vulnerable to possible cytotoxic effects of vancomycin. This might be caused by differences in the biologic microstructure of both graft types and may also be related to the protective influence of the intact tendo-synovial sheath after HT autograft harvesting when compared to QT autograft harvesting: usually, the tendon sheath remains intact after HT autograft harvesting but not after QT autograft harvesting [23, 26]. Direct exposure of QT fibers to vancomycin may cause violation of the tendon graft [27]. Further large-scale clinical and in vitro studies are warranted to carefully assess possible cytotoxic effects of vancomycin on QT tendon autografts.

## Limitations of the Study

The main limitation of this investigation is its non-randomized and non-blinded design. The unequal group sizes (HT = 421 vs QT = 47) represent another limitation that may have influenced statistical power. Furthermore, we cannot reliably attribute early graft failure specifically to vancomycin use, as vancomycin was not assessed as an independent variable and other confounding factors may have contributed to the observed outcome. Therefore, a randomized controlled trial comparing QT autograft ACLR with and without vancomycin would be required to confirm our findings. We also acknowledge that some patients underwent their primary ACLR at other institutions, which may have introduced minor variations in perioperative management or immediate postoperative care, even though these are unlikely to have affected the overall results. This consideration also applies to the postoperative rehabilitation protocol. In addition, no histological or molecular analyses were performed.

## Conclusion

The markedly higher rate of early graft failure due to graft resorption observed in the QT group raises concern regarding the safety of combining vancomycin soaking with quadriceps tendon autografts for ACL reconstruction. Given the proven infection-preventive benefit of vancomycin and the lack of data specifically addressing QT autografts, these findings should be interpreted with caution and considered hypothesis-generating. Until further evidence becomes available, surgeons should exercise caution when using vancomycin-soaked QT autografts, while the combination of hamstring tendon autografts with vancomycin remains the more established and better-studied option. Prospective clinical and experimental studies are needed to confirm these observations and clarify the underlying mechanisms.
